# (η^4^-*s*-*cis*-1,3-Butadiene)tetra­carbonyl­chromium(0)

**DOI:** 10.1107/S1600536811004636

**Published:** 2011-02-12

**Authors:** Guido J. Reiss, Maik Finze

**Affiliations:** a Institut für Anorganische Chemie und Strukturchemie, Lehrstuhl II: Material- und Strukturforschung, Heinrich-Heine-Universität Düsseldorf, Universitätsstrasse 1, D-40225 Düsseldorf, Germany

## Abstract

In the title complex, [Cr(C_4_H_6_)(CO)_4_], the Cr^0^ atom shows a distorted octa­hedral environment from four C atoms of the carbonyl ligands and the two π-bonds of the *s-cis*-1,3-butadiene ligand. The complex has an approximate non-crystallographic mirror symmetry *m* passing through the chromium atom, two carbonyl ligands and the mid-point of the central C—C bond of the *s-cis*-1,3-butadiene ligand. The C—C bond lengths in the *s-cis*-1,3-butadiene ligand alternate, the terminal distances being shorter than the central distance.

## Related literature

For experimental and theoretical data for the title compound, see: Fischler *et al.* (1976 ▶); Kotzian *et al.* (1982 ▶); Kreiter & Özkar (1978 ▶); Okamoto *et al.* (1991 ▶); von Ragué Schleyer *et al.* (2000 ▶). For related chromium complexes, see: Pavkovic & Zaluzec (1989 ▶), Betz *et al.* (1993 ▶), Wang *et al.* (1990 ▶), Konietzny *et al.* (2010 ▶). For related s-*cis*-butadiene complexes, see: Reiss (2010 ▶), Reiss & Konietzny (2002 ▶).
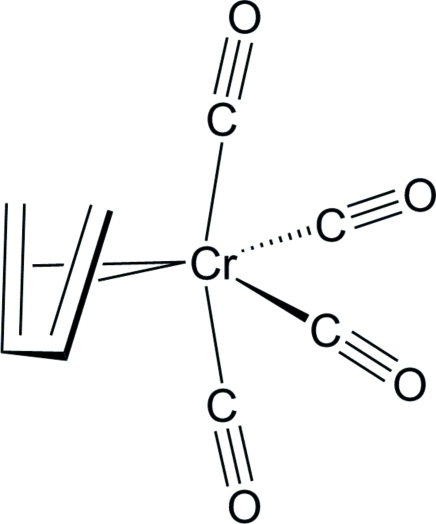


## Experimental

### 

#### Crystal data

[Cr(C_4_H_6_)(CO)_4_]
*M* *_r_* = 218.13
Triclinic, 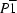

*a* = 6.4011 (8) Å
*b* = 6.7666 (8) Å
*c* = 11.0642 (10) Å
α = 84.728 (7)°
β = 81.840 (8)°
γ = 69.127 (8)°
*V* = 442.80 (8) Å^3^
*Z* = 2
Mo *K*α radiation
μ = 1.27 mm^−1^
*T* = 137 K
0.38 × 0.26 × 0.04 mm

#### Data collection

Oxford Diffraction Xcalibur Eos diffractometer
Absorption correction: Gaussian (*CrysAlis PRO*; Oxford Diffraction, 2009 ▶) *T*_min_ = 0.711, *T*_max_ = 0.946
2829 measured reflections
1735 independent reflections
1498 reflections with *I* > 2σ(*I*)
*R*_int_ = 0.020
3 standard reflections every 60 min  intensity decay: none

#### Refinement

*R*[*F* ^2^ > 2σ(*F* ^2^)] = 0.028
*wR*(*F* ^2^) = 0.068
*S* = 1.05
1735 reflections
140 parameters
All H-atom parameters refined
Δρ_max_ = 0.29 e Å^−3^
Δρ_min_ = −0.38 e Å^−3^

### 

Data collection: *CrysAlis PRO* (Oxford Diffraction, 2009 ▶); cell refinement: *CrysAlis PRO*; data reduction: *CrysAlis PRO*; program(s) used to solve structure: *SHELXS97* (Sheldrick, 2008 ▶); program(s) used to refine structure: *SHELXL97* (Sheldrick, 2008 ▶); molecular graphics: *DIAMOND* (Brandenburg, 2010 ▶); software used to prepare material for publication: *SHELXL97*.

## Supplementary Material

Crystal structure: contains datablocks I, global. DOI: 10.1107/S1600536811004636/si2332sup1.cif

Structure factors: contains datablocks I. DOI: 10.1107/S1600536811004636/si2332Isup2.hkl

Additional supplementary materials:  crystallographic information; 3D view; checkCIF report

## Figures and Tables

**Table 1:** Selected bond lengths (Å)

Cr1—C5	1.852 (2)
Cr1—C6	1.887 (2)
Cr1—C7	1.873 (2)
Cr1—C8	1.914 (2)
Cr1—C1	2.312 (2)
Cr1—C2	2.184 (2)
Cr1—C3	2.190 (2)
Cr1—C4	2.325 (2)
C1—C2	1.379 (3)
C2—C3	1.436 (3)
C3—C4	1.371 (3)

**Fig. 1.**
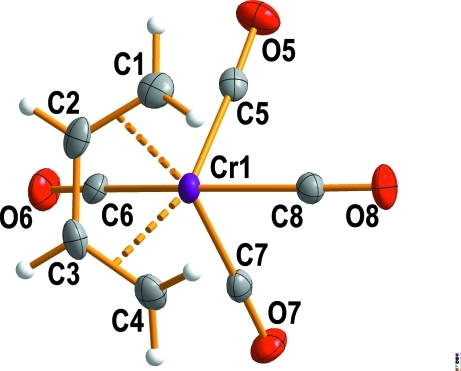
Hydrogen atoms are drawn with an arbitrary radius and the displacement ellipsoids are shown at the 50% probability level.

## supplementary crystallographic information

### Comment

Simple butadiene complexes of transition metals are of general interest because they are model systems that allow a deeper understanding of the bonding situation between transition metal centers and olefins that play an important role for example in catalysis. [Cr(C_4_H_6_)(CO)_4_] that was first described in the 70s of the last century (Fischler *et al.* 1976) was subject to a number of spectroscopic (Kotzian *et al.* 1982) as well as theoretical studies (von Ragué Schleyer *et al.* 2000) and its chemistry was investigated (Kreiter & Özkar, 1978; Okamoto *et al.* 1991) with the focus on photochemical ligand exchange reactions (Fischler *et al.* 1976).

The coordination at Cr(0) in the title compound is best described as a distorted octahedron formed by four carbonyl ligands and one *s*-*cis*-1,3-butadiene ligand. The Cr–CO distances of the carbonyl ligands that are *trans* to the *s*-*cis*-1,3-butadiene ligand are slightly shorter than the two other Cr–CO distances (Table 1). This finding is in good agreement to Cr—CO distances in the structure of the related tetracarbonyl chromium(0) complex [Cr(C_19_H_23_NO_2_)(CO)_4_]: *d*(Cr–CO*_trans_*) = 1.884 (4), 1.887 (6) Å and *d*(Cr–CO) = 1.847 (5), 1.837 (4) Å (Pavkovic & Zaluzec, 1989). In the structure of the title complex the Cr–C distances to the terminal carbon atoms of the *s*-*cis*-1,3-butadiene ligand are longer compared to the respective distances to the central carbon atoms of the diene ligand. A similar trend to longer Cr–C distances for the terminal carbon atoms was found for example for the *s*-*cis*-1,3-butadiene chromium(1) complex [CrCp*(C_4_H_6_)(CO)] (Betz *et al.* 1993). As known from a few other chromium(0) complexes of *s*-*cis*-1,3-butadiene and related coordination compounds (Pavkovic & Zaluzec, 1989; Betz *et al.* 1993; Wang *et al.* 1990; Konietzny *et al.* 2010) in [Cr(C_4_H_6_)(CO)_4_] the terminal C–C distances are significantly shorter than the central *d*(C–C) Δ(*d*(C–C)) = 0.057–0.065 Å. In contrast, for comparable iron(0) and manganese(0) complexes almost equilibrated C–C distances have been reported (Reiss, 2010; Reiss & Konietzny 2002), e. g. in the structure of the *s*-*cis*-1,3-butadiene iron(0) complex [Fe(C_4_H_6_)(CO)_3_] Δ(*d*(C–C)) = 0.005 Å [*d*(C–C)_central_ = 1.4142 (19) Å, *d*(C–C)_terminal_ = 1.4194 (14) Å] (Reiss, 2010).

### Experimental

Synthesis

[Cr(C_4_H_6_)(CO)_4_] was synthesized according to a published procedure (Fischler, 1976). The crystal was obtained by slow evaporation of a solution of pentane.

### Refinement

All hydrogen atoms were located from difference Fourier synthesis. For the terminal H atom pairs of the CH_2_ groups common *U*_iso_(H) = 0.031 (4)/0.027 (4) Å^2^ and individual *U*_iso_(H) = 0.027 (6) and 0.019 (5) Å^2^ for the two central H atoms were refined freely with distances in the range 0.90 (2) - 0.98 (3) Å.

### Crystal data

**Table:**

[Cr(C_4_H_6_)(CO)_4_]	*Z* = 2
*M**_r_* = 218.13	*F*(000) = 220
Triclinic, *P*1	*D*_x_ = 1.636 Mg m^−^^3^
Hall symbol: -P 1	Mo *K*α radiation, λ = 0.71073 Å
*a* = 6.4011 (8) Å	Cell parameters from 2257 reflections
*b* = 6.7666 (8) Å	θ = 3.4–28.7°
*c* = 11.0642 (10) Å	µ = 1.27 mm^−^^1^
α = 84.728 (7)°	*T* = 137 K
β = 81.840 (8)°	Platelet, yellow
γ = 69.127 (8)°	0.38 × 0.26 × 0.04 mm
*V* = 442.80 (8) Å^3^	

### Data collection

**Table:**

Oxford Diffraction Xcalibur Eos diffractometer	1498 reflections with *I* > 2σ(*I*)
Radiation source: fine-focus sealed tube	*R*_int_ = 0.020
graphite	θ_max_ = 26.0°, θ_min_ = 4.1°
ω scans	*h* = −7→7
Absorption correction: gaussian (*CrysAlis PRO*; Oxford Diffraction, 2009)	*k* = −8→8
*T*_min_ = 0.711, *T*_max_ = 0.946	*l* = −13→13
2829 measured reflections	3 standard reflections every 60 min
1735 independent reflections	intensity decay: none

### Refinement

**Table:**

Refinement on *F*^2^	Primary atom site location: structure-invariant direct methods
Least-squares matrix: full	Secondary atom site location: difference Fourier map
*R*[*F*^2^ > 2σ(*F*^2^)] = 0.028	Hydrogen site location: inferred from neighbouring sites
*wR*(*F*^2^) = 0.068	All H-atom parameters refined
*S* = 1.05	*w* = 1/[σ^2^(*F*_o_^2^) + (0.04*P*)^2^] where *P* = (*F*_o_^2^ + 2*F*_c_^2^)/3
1735 reflections	(Δ/σ)_max_ = 0.001
140 parameters	Δρ_max_ = 0.29 e Å^−^^3^
0 restraints	Δρ_min_ = −0.38 e Å^−^^3^

### Special details

**Table:**

Experimental. A single-crystal suitable for structure determination was harvested under a dry nitrogen atmosphere and was directly transferred into the cooling stream of an Oxford-Xcalibur diffractometer equipped with an EOS-CCD detector. *CrysAlis PRO*, Oxford Diffraction Ltd., Version 1.171.33.52 (release 06–11-2009). Numerical absorption correction based on Gaussian integration over a multifaceted crystal model.
Geometry. All e.s.d.'s (except the e.s.d. in the dihedral angle between two l.s. planes) are estimated using the full covariance matrix. The cell e.s.d.'s are taken into account individually in the estimation of e.s.d.'s in distances, angles and torsion angles; correlations between e.s.d.'s in cell parameters are only used when they are defined by crystal symmetry. An approximate (isotropic) treatment of cell e.s.d.'s is used for estimating e.s.d.'s involving l.s. planes.
Refinement. Refinement of *F*^2^ against ALL reflections. The weighted *R*-factor *wR* and goodness of fit *S* are based on *F*^2^, conventional *R*-factors *R* are based on *F*, with *F* set to zero for negative *F*^2^. The threshold expression of *F*^2^ > σ(*F*^2^) is used only for calculating *R*-factors(gt) *etc*. and is not relevant to the choice of reflections for refinement. *R*-factors based on *F*^2^ are statistically about twice as large as those based on *F*, and *R*- factors based on ALL data will be even larger.

### Fractional atomic coordinates and isotropic or equivalent isotropic displacement parameters (Å^2^)

**Table:**

	*x*	*y*	*z*	*U*_iso_*/*U*_eq_	
Cr1	0.66351 (5)	0.59871 (5)	0.74506 (3)	0.01600 (12)	
O6	0.8591 (3)	0.2852 (3)	0.94893 (13)	0.0342 (4)	
C6	0.7852 (3)	0.4125 (3)	0.87540 (17)	0.0212 (4)	
O5	0.3023 (3)	0.4055 (2)	0.79702 (16)	0.0342 (4)	
O8	0.4722 (3)	0.7542 (3)	0.50255 (13)	0.0329 (4)	
C8	0.5444 (3)	0.7032 (3)	0.59328 (18)	0.0222 (4)	
C5	0.4434 (3)	0.4763 (3)	0.77460 (18)	0.0216 (4)	
O7	1.0248 (3)	0.2629 (3)	0.59598 (14)	0.0329 (4)	
C7	0.8870 (3)	0.3908 (3)	0.65151 (18)	0.0222 (4)	
C3	0.8024 (4)	0.8098 (3)	0.81772 (19)	0.0258 (5)	
H3	0.921 (4)	0.746 (4)	0.864 (2)	0.027 (6)*	
C2	0.5762 (4)	0.8487 (3)	0.8746 (2)	0.0269 (5)	
H2	0.556 (3)	0.811 (3)	0.955 (2)	0.019 (5)*	
C1	0.3978 (4)	0.9184 (4)	0.8059 (2)	0.0287 (5)	
H12	0.403 (4)	1.009 (4)	0.732 (2)	0.031 (4)*	
H11	0.261 (4)	0.918 (4)	0.846 (2)	0.031 (4)*	
C4	0.8493 (4)	0.8394 (4)	0.6940 (2)	0.0282 (5)	
H41	0.748 (4)	0.940 (4)	0.647 (2)	0.027 (4)*	
H42	0.995 (4)	0.789 (4)	0.655 (2)	0.027 (4)*	

### Atomic displacement parameters (Å^2^)

**Table:**

	*U*^11^	*U*^22^	*U*^33^	*U*^12^	*U*^13^	*U*^23^
Cr1	0.01916 (18)	0.01729 (18)	0.01228 (17)	−0.00607 (13)	−0.00425 (11)	−0.00170 (12)
O6	0.0401 (9)	0.0363 (10)	0.0225 (8)	−0.0080 (8)	−0.0115 (7)	0.0083 (7)
C6	0.0233 (10)	0.0242 (11)	0.0161 (10)	−0.0078 (9)	−0.0006 (8)	−0.0052 (9)
O5	0.0282 (8)	0.0266 (9)	0.0510 (10)	−0.0139 (7)	−0.0031 (7)	−0.0027 (8)
O8	0.0432 (9)	0.0350 (9)	0.0230 (8)	−0.0128 (8)	−0.0179 (7)	0.0042 (7)
C8	0.0234 (10)	0.0225 (11)	0.0223 (11)	−0.0089 (9)	−0.0038 (8)	−0.0034 (9)
C5	0.0233 (10)	0.0165 (10)	0.0222 (10)	−0.0017 (9)	−0.0065 (8)	−0.0023 (8)
O7	0.0317 (9)	0.0334 (9)	0.0260 (8)	−0.0027 (7)	0.0040 (7)	−0.0098 (7)
C7	0.0258 (11)	0.0263 (11)	0.0171 (10)	−0.0113 (9)	−0.0066 (8)	0.0025 (9)
C3	0.0335 (12)	0.0233 (11)	0.0266 (11)	−0.0133 (10)	−0.0142 (9)	−0.0010 (9)
C2	0.0429 (13)	0.0194 (11)	0.0200 (11)	−0.0111 (10)	−0.0045 (9)	−0.0073 (9)
C1	0.0309 (12)	0.0188 (11)	0.0339 (13)	−0.0047 (9)	−0.0013 (10)	−0.0080 (10)
C4	0.0312 (13)	0.0305 (13)	0.0302 (12)	−0.0189 (11)	−0.0080 (10)	0.0021 (10)

### Geometric parameters (Å, °)

**Table:**

Cr1—C5	1.852 (2)	O8—C8	1.138 (2)
Cr1—C6	1.887 (2)	C1—C2	1.379 (3)
Cr1—C7	1.873 (2)	C2—C3	1.436 (3)
Cr1—C8	1.914 (2)	C3—C4	1.371 (3)
Cr1—C1	2.312 (2)	C1—H11	0.92 (2)
Cr1—C2	2.184 (2)	C1—H12	0.98 (3)
Cr1—C3	2.190 (2)	C2—H2	0.90 (2)
Cr1—C4	2.325 (2)	C3—H3	0.92 (2)
O5—C5	1.153 (3)	C4—H41	0.93 (3)
O6—C6	1.148 (3)	C4—H42	0.93 (3)
O7—C7	1.142 (3)		
			
C5—Cr1—C6	83.10 (9)	C2—C3—C4	121.6 (2)
C5—Cr1—C7	99.88 (9)	C2—C1—H11	116.0 (15)
C7—Cr1—C6	82.30 (8)	C2—C1—H12	120.2 (14)
C5—Cr1—C8	85.80 (9)	C1—C2—H2	120.7 (14)
C7—Cr1—C8	84.94 (9)	C3—C2—H2	118.0 (14)
C6—Cr1—C8	161.38 (9)	C4—C3—H3	118.6 (15)
O6—C6—Cr1	174.03 (18)	C2—C3—H3	119.2 (15)
O8—C8—Cr1	176.01 (19)	C3—C4—H41	122.7 (15)
O5—C5—Cr1	177.16 (18)	C3—C4—H42	121.8 (15)
O7—C7—Cr1	178.99 (18)	H12—C1—H11	120 (2)
C1—C2—C3	120.8 (2)	H41—C4—H42	114 (2)
			
C4—C3—C2—C1	−0.3 (3)		

## References

[bb1] Betz, P., Döhring, A., Emrich, R., Goddard, R., Jolly, P. W., Krüger, C., Romão, C. C., Schönfelder, K. U. & Tsay, Y.-H. (1993). *Polyhedron*, **12**, 2651–2662.

[bb2] Brandenburg, K. (2010). *DIAMOND* Crystal Impact GbR, Bonn, Germany.

[bb3] Fischler, M., Budzwait, M. & Koerner von Gustorf, E. A. (1976). *J. Organomet. Chem.* **105**, 325–330.

[bb4] Konietzny, S., Finze, M. & Reiss, G. J. (2010). *J. Organomet. Chem.* **695**, 2089–2092.

[bb5] Kotzian, M., Kreiter, C. G. & Özkar, S. (1982). *J. Organomet. Chem.* **229**, 29–42.

[bb6] Kreiter, C. G. & Özkar, S. (1978). *J. Organomet. Chem.* **152**, C13–C18.

[bb7] Okamoto, Y., Inui, Y., Onimatsu, H. & Imanaka, T. (1991). *J. Phys. Chem.* **95**, 4596–4598.

[bb8] Oxford Diffraction (2009). *CrysAlis PRO* Oxford Diffraction Ltd, Yarnton, England.

[bb9] Pavkovic, S. F. & Zaluzec, E. J. (1989). *Acta Cryst.* C**45**, 18–21.

[bb10] Ragué Schleyer, P. von, Kiran, B., Simion, D. V. & Sorensen, T. S. (2000). *J. Am. Chem. Soc.* **122**, 510–513.

[bb11] Reiss, G. J. (2010). *Acta Cryst.* E**66**, m1369. 10.1107/S1600536810039218 PMC3009352 21588810

[bb12] Reiss, G. J. & Konietzny, S. (2002). *Dalton Trans.* pp. 862–864.

[bb13] Sheldrick, G. M. (2008). *Acta Cryst.* A**64**, 112–122. 10.1107/S0108767307043930 18156677

[bb14] Wang, N.-F., Wink, D. J. & Dewan, J. C. (1990). *Organometallics*, **9**, 335–340.

